# Limited Utility of ITPA Deficiency to Predict Early Anemia in HCV Patients with Advanced Fibrosis Receiving Telaprevir

**DOI:** 10.1371/journal.pone.0095881

**Published:** 2014-04-23

**Authors:** Alessio Aghemo, Eleonora Grassi, Maria Grazia Rumi, Roberta D'Ambrosio, Enrico Galmozzi, Elisabetta Degasperi, Davide Castaldi, Roberta Soffredini, Massimo Colombo

**Affiliations:** 1 A.M. and A. Migliavacca Center for Liver Disease, First Division of Gastroenterology, Fondazione IRCCS Cà Granda Ospedale Maggiore Policlinico, Università degli Studi di Milano, Milan, Italy; 2 Hepatology Unit, Ospedale San Giuseppe, IRCCS Multimedica, Università degli Studi di Milano, Milan, Italy; 3 Department of Informatics, Systemistic and Comunication (DISCo), Universita' degli Studi di Milano – Bicocca, Milan, Italy; Centers for Disease Control and Prevention, United States of America

## Abstract

**Background:**

Severe anemia is a common side effect of Pegylated Interferon + Ribavirin (PR) and Telaprevir (TVR) in hepatitis C virus (HCV) genotype 1 patients with advanced fibrosis or cirrhosis (F3–F4). Inosine triphosphatase (ITPA) genetic variants are associated with RBV- induced anemia and dose reduction.

**Aim:**

To test the association of ITPA polymorphisms rs1127354 and rs7270101 with hemoglobin (Hb) decline, need for RBV dose reduction (RBV DR), erythropoietin (EPO) support and blood transfusions during the first 12 weeks of TVR triple therapy.

**Materials and Methods:**

69 consecutive HCV-1 patients (mean age 57 years) with F3-F4 who received PR and TVR were genotyped for ITPA polymorphisms *rs1127354* and *rs7270101*. Estimated ITPA deficiency was graded on severity (0–3, no deficiency/mild/moderate/severe).

**Results:**

ITPA deficiency was absent in 48 patients (70%), mild in 12 (17%) and moderate in 9 patients (13%). Mean week 4 Hb decline was higher in non ITPA deficient patients (3,85 g/dL) than in mildly or moderately ITPA deficient patients (3,07 g/dL and 1,67 g/dL, p<0.0001). Grade 3–4 anemia developed in 81% non ITPA deficient patients versus 67% mild deficient and 55% moderate deficient patients (p = ns). Grade of ITPA deficiency was not associated with RbvDR (no deficiency: 60%, mild: 58%, moderate: 67%; p = ns), EPO use (no deficiency: 65%, mild: 58%, moderate:56%; p = ns) or need for blood transfusion (no deficiency: 27%, mild: 17%, moderate: 33%; p = ns).

**Conclusions:**

In patients with F3–F4 chronic hepatitis C receiving TVR based therapy, ITPA genotype does not impact on the management of early anemia.

## Introduction

The first generation inhibitors of the Hepatitis C virus (HCV) NS3 protein, Telaprevir (TVR) and Boceprevir (BOC), when combined with Pegylated Interferon and Ribavirin (PR) are the standard of care regimen for patients with HCV-1 [1]. This triple therapy regimen has provided an increase in achievable sustained virological response (SVR) rates compared to PR both in treatment naive patients, as well as in patients who have failed to respond to a previous course of PR. Still in the most in need group of patients, those with advanced fibrosis/cirrhosis, SVR rates are still subpar, especially in the subgroup of those classified as null-responders to the previous course of PR [2]. Moreover, several real-life studies have shown that in patients with advanced fibrosis/cirrhosis the side effect profile of triple therapy is especially cumbersome in terms of anemia and infection rates, often leading to premature treatment discontinuation or development of serious adverse events (SAE) [3]–[4]. With regards to TVR, the international early access program, enrolling more than 1600 patients with advanced fibrosis/cirrhosis who received PR+TVR at standard dose, demonstrated that on treatment anemia developing in the first 12–16 weeks is the main clinical issue of this regimen [4]. Indeed anemia was the most common SAE and the most frequent reason for treatment discontinuation, ultimately requiring blood transfusions in 10% of the patients and Erythropoietin support in nearly 20% of enrolled patients. This data is consistent with other real-life studies of TVR, reporting anemia to be the most frequent cause for SAE [5]–[6]. Baseline prediction of early anemia to TVR based regimens in patients with advanced fibrosis/cirrhosis is therefore of paramount importance as it may allow individual counselling and timely interventions to correctly manage this side effect.

For this reason we decided to study the role of single nucleotide polymorphisms (SNPs) in the Inosine Triphosphatase (ITPA) gene in determining PR+TVR early anemia in patients with HCV-1 advanced fibrosis/cirrhosis. The combination of the two ITPA SNPs, rs1127354 and rs7270101, determines the relative deficiency of the ITPA protein, which in turn has been associated with the grade of anemia during PR therapy [7]–[8]. The explained mechanism is that RBV-metabolites accumulate within erythrocytes depleting intracellular adenosine triphosphate (ATP) and guanosine triphosphate (GTP), leading to membrane oxidative stress and consequent haemolysis. The reduced ITPA activity increases intracellular Inosine triphosphate (ITP) levels, allowing ITP to substitute GTP in the generation of Adenosine monophosphate (AMP), thus maintaining the activity of membrane transporters and avoiding haemolysis of red cells.

However the clinical utility of ITPA SNPs in predicting anemia severity in TVR based regimens to date is still relatively unknown, as it has been studied mostly in patients of Asian ethnicity and never in patients with advanced fibrosis/cirrhosis [9]–[10].

## Patients and Methods

### Patients

This retrospective cohort study was performed in all consecutive HCV-1 patients with chronic hepatitis C genotype 1 and advanced fibrosis who received TVR based therapy at the Liver Center of the Ospedale Maggiore Policlinico in Milan and at the Hepatology Unit of the San Giuseppe Hospital in Milan between January and June 2013. All patients gave their written consent for treatment and genotyping of ITPA polymorphisms *rs1127354* and *rs7270101*.

Chronic hepatitis C was defined by at least one year serum positivity for serum HCV-RNA assessed by quantitative RT-PCR assay with detection limit of 12 IU/ml. Advanced Fibrosis was defined by a liver biopsy performed before treatment, consistent with chronic hepatitis C and F3–F4 according to the METAVIR score, or by a transient elastography value ≥9.5 KPa. Fibroscan cut-off to diagnose bridging fibrosis and cirrhosis was ≥9.5 KPa and ≥12.5 KPa, respectively.

Exclusion criteria were: 1) co-infection with hepatitis B virus (HBV) or human immunodeficiency virus (HIV), 2) decompensated liver disease, 3) drug dependence or >40 g/day alcohol intake, 4) general contraindication to PegIFN and Rbv treatment, 5) pre-existing anemia due to hemoglobin disorders, 6) malignancy. The study was approved by the IRB of the Department of Medicine of the Ospedale Maggiore Policlinico of Milan.

### Antiviral Treatment

All patients received a combination of TVR 750 mg every 8 hours, PegIFNα2a 180 mcg/week or PegIFNα2b 1.5 mcg/Kg/week, and Rbv for 12 weeks, followed by an additional 36 weeks of PegIFN and Rbv alone. Treatment duration followed the EMA approved TVR label. Telaprevir was administrated every 8 hours with food. Rbv was administrated according to PegIFN label. All patients were evaluated for safety and tolerance of therapy every 2 weeks during the TVR treatment period. PegIFNα2a was reduced to 135 mcg and PegIFNα2b to 1,0 mcg/Kg per week in patients with <0,75×10^9^/L neutrophils at two consecutive tests whereas it was interrupted in patients with <0,50×10^9^/L. The same dose reductions were applied if platelets were under 50,000 cells/mm^3^ with PegIFN being discontinued when reaching the 25,000 cells/mm^3^ threshold.

Therapy was discontinued if HCV-RNA was >1000 IU/ml at week 4 or 12, if HCV-RNA was detectable at week 24 and in case of virological breakthrough (any detectable HCV RNA after achieving undetectable levels).

### Menagement of anemia

Complete blood count was checked every 2 weeks for the first 12 weeks of therapy, and then every 4 weeks. Anemia severity was defined as grade 1 when Hb values were between 10–10,9 g/dl or Hb decline was 2,5–3,4 g/dl from baseline; grade 2 anemia was Hb values between 9,0–9,9 g/dl or Hb decline of 3,5–4,4 g/dl from baseline, grade 3 anemia was Hb between 7,0–8,9 g/dl or Hb decline>4,5 from baseline; grade 4 for was Hb<7,0 g/dl.

Rbv dose reductions (RbvDR), growth factors and blood transfusions were allowed for the management of anemia. The management strategy was at the discretion of the investigator, however Rbv dose reductions and/or Erythropoietin alfa 20,000 to 40,000 IU/week administration were allowed to manage anemia only if Hb levels were less than 10 g/dL. Blood transfusions were allowed only if Hb was <8,5 g/dl. If Rbv had to be discontinued, TVR was discontinued at the same time and the decision to continue PegIFN monotherapy or discontinue treatment was at the discretion of the investigator.

### Measurements

Serum HCV-RNA was assessed by the Abbott RT-PCR assay with a detection limit of 12 IU/ml at weeks 2, 4, 8 and 12. Liver biopsies were performed with a 16 gauge Tru-Cut needle (Uro-Cut 16G, TSK, Tokyo, Japan) and read by a single pathologist who was unaware of patient's identity and treatment regimen. Liver biopsies fibrosis stage was assessed according to the METAVIR scoring system [11]: F3 numerous septa without cirrhosis, F4 cirrhosis.

Liver stiffness measurement (LSM) was performed by fibroscan as already described [12]. LSM was expressed in kiloPascal (kPa) as the median value of the successful measurements. Only LSM data with at least ten successful measurements, success rate higher than 60%, and inter quartile ratio (IQR) inferior to 30%, were considered reliable [13]. LSM were performed by officially trained operators.

### Genetic testing

Genotyping of the ITPA polymorphisms *rs1127354* and *rs7270101* was performed using the Allelic Inhibition of Displacement Activity (AIDA) assay [14]. For the ITPA *rs1127354* polymorphism heterozygotes (CA) or homozygotes (AA) of the minor allele (A) are described as having the ITPA minor allele, whereas homozygotes for the major allele (CC) are described as having the ITPA major allele.

Conversely for the ITPA *rs7270101* polymorphism heterozygotes (AC) or homozygotes (CC) of the minor allele (C) are described as having the ITPA minor allele, whereas homozygotes for the major allele (AA) are described as having the ITPA major allele. ITPA deficiency phenotypes were then classified according to the degree of predicted ITPA deficiency (0–3: no ITPA deficiency, mild, moderate or severe deficiency), based on the combination of bi-allelic polymorphisms (table 1).

**Table 1:** Degrees of ITPA deficiency according to rs1127354/rs7270101 genotypes combinations (0–3: no ITPA deficiency, mild, moderate or severe deficiency).

rs1127354	rs7270101	Predicted ITPA activity	Predicted ITPA deficiency
Wild-type (C/C)	Wild-type (A/A)	100%	0
Wild-type (C/C)	Heterozygosity (A/C)	60%	1
Heterozygosity (C/A)	Wild-type (A/A)	30%	2
Wild-type (C/C)	Homozygosity (C/C)	30%	2
Heterozygosity (C/A)	Heterozygosity (A/C)	10%	3
Homozygosity (A/A)	Wild-type (A/A)	<5%	3

### Statistical analysis

Statistical analyses were conducted using the Mann–Whitney U test or the Student t test for continuous variables and the χ2 or Fisher exact probability test for categorical data. A probability value of p<0,05 was considered statistically significant. All variables with statistical significance at the univariate analaysis were included in the final model and odds ratios (OR) and corresponding 95% confidence interval (95% CI) were computed. Calculations were done with Stata 10.0 statistical package.

## Results

### Patients

During the enrolment period 69 patients met the inclusion criteria and were analyzed in the present study. Patients characteristics are shown in table 2. The mean age was 57 years old, with 46% older than 60 years. Female patients represented 33% of the overall cohort. All patients had advanced fibrosis and 51 (74%) had cirrhosis, in 28 cases defined by liver biopsy and in the remaining 23 cases by TE. No patient had pre-treatment anemia or impaired baseline kidney function, the mean estimated glomerular filtration rate was 93 ml/min/1,73 m^2^ by the MDRD equation. HCV genotype 1b was the most frequent subtype being found in 83% of the patients. Only 9 (13%) patients were treatment naïve, the remaining 60 treatment experienced patients were classified as relapsers (38%), partial responders (14%), null responders (32%) or virological breakthroughs (3%) to a previous PR treatment. 48 patients (70%) had no ITPA deficiency, the remaining 21 (30%) showed varying degrees of deficiency: mild ITPA deficiency was found in 12 (17%) and moderate deficiency was found in 9 patients (13%). None of the enrolled patients showed severe ITPA deficiency. Epidemiological, clinical and virological features did not differ between ITPA deficient and non deficient patients (data not shown).

**Table 2:** Patients characteristics.

Feature	Overall
**Female sex, n(%)**	23 (33%)
**Age years, mean (range)**	57 (35–69)
**Cirrhosis, n(%)**	51 (74%)
**eGFR, mean ml/min**	93,2
**HCV 1b genotype, n(%)**	57 (83%)
**HCV-RNA>600.000 IU/ml, n(%)**	42 (61%)
**Hb g/dL, mean (range)**	15,1 (10,8–18,0)
Male	15,6 (12,0–18,0)
Female	14,2 (10,8–15,9)
**Previous treatment status, n(%)**	
Naive	9 (13%)
Relapse	26 (38%)
Partial Response	10 (14%)
Null response	22 (32%)
Breakthrough	2 (3%)
**Grade of ITPA deficiency, n(%)**	
No deficiency	48 (70%)
Mild deficiency	12 (17%)
Moderate deficiency	9 (13%)

### ITPA deficiency and severity of anemia

57 patients (83%) completed the 12 weeks of PR+TVR treatment. The reasons from premature treatment discontinuation in the remaining 12 patients were the following: virological stopping rule in 2 cases (3%), severe anemia in 5 (7%), Hepatocellular carcinoma (HCC) in 1 patient (1%) and treatment related side effects other than anemia in 4 cases (6%). Treatment discontinuation rates were 21% (10/48) of non ITPA deficient patients, in 8% (1/12) of mild and 11% (1/9) of moderate deficient patients (p = 0.3). Premature discontinuation for severe anemia occurred in 8% of non ITPA deficient patients (4/48), 8% of mild deficient patients (1/12) and 0% of moderate deficient patients (p = 1).

Any grade anemia was observed in 68 (98%) patients: 2 (3%) patients developed grade 1 anemia, 14 (20%) developed grade 2 anemia, 51 (74%) developed grade 3 anemia and 1 (1%) developed grade 4 anemia. Grade 3–4 anemia developed in 81% of non ITPA deficient versus 67% of mild ITPA deficient patients and 55% of moderate ITPA deficient patients (p = 0.1). The only factor associated with development of grade 3–4 anemia was age >60 years (OR 5,88; 95% CI: 1,50–22,96).

**Figure 1:**
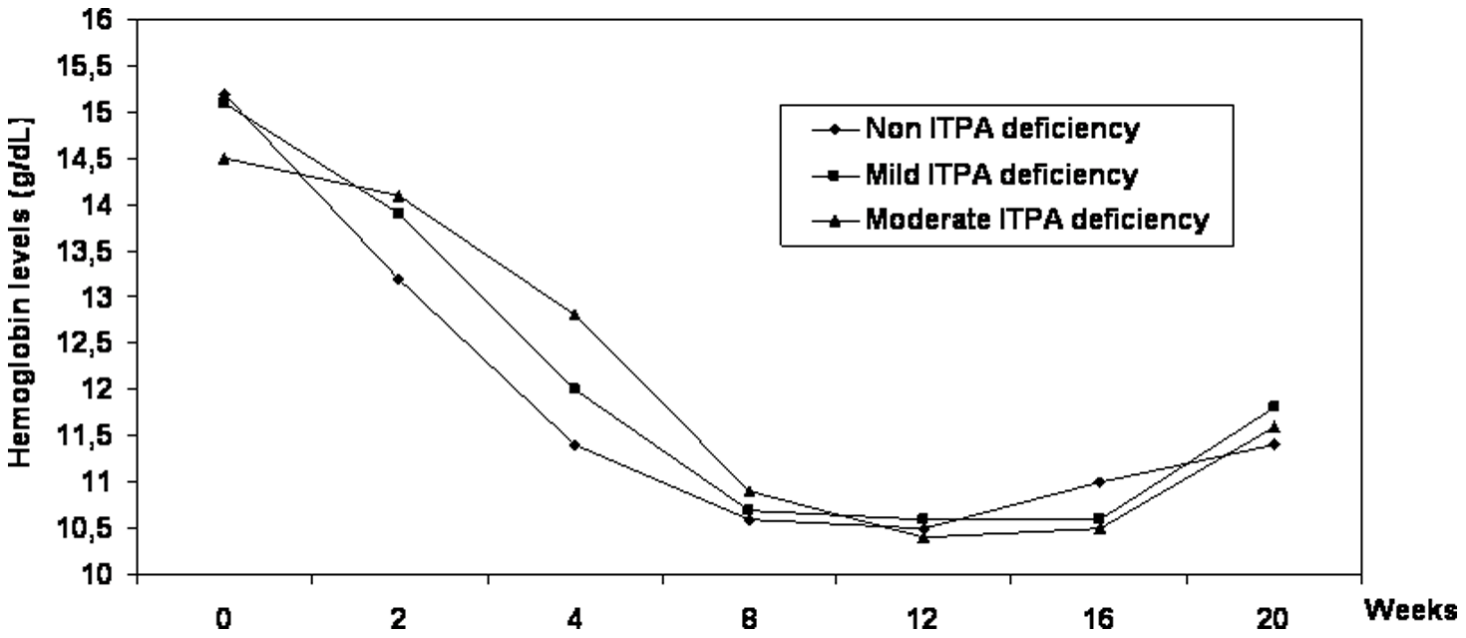
Mean hemoglobin values during treatment with PR+TVR stratified by ITPA deficiency.

The mean Hb values during the first 16 weeks of treatment stratified by ITPA deficiency are shown in figure 1. The mean decline in Hb values after 2 and 4 weeks of treatment was significantly more pronounced in patients without ITPA deficiency than in mild ITPA deficient patients and moderate ITPA deficient patients (week 2 Hb decline: 1,99 g/dl vs 1,26 g/dl vs 0,37 g/dl, p = 0,0006; week 4 Hb decline: 3,85 g/dl vs 3,07 g/dl vs 1,67 g/dl, p<0,0001). This resulted in significantly lower mean Hb values after 4 weeks of PR+TVR in patients without ITPA deficiency compared to patients with mild or moderate ITPA deficiency (week 4 Hb: 11,4 g/dl vs 12,4 g/dl, p = 0,01).

However due to a sharper decline in Hb values at week 8 and 12 of PR+TVR in ITPA deficient patients, the week 8 and 12 levels of Hb did not differ across ITPA deficient categories (figure 1).

At the time of analysis, 42 and 32 patients respectively have undergone blood tests at week 16 and 20 of therapy, i.e 4 and 8 weeks after discontinuation of TVR. Hb levels at these time points did not significantly differ between ITPA deficient and non deficient patients (figure 1).

### ITPA deficiency and management of anemia

During the first 12 weeks of PR+TVR, 42 patients (60%) required RbvDR. RbvDR rates were not associated with ITPA deficiency as they occurred in 60% of ITPA non deficient patients, in 58% mild ITPA deficient patients and 67% moderate ITPA deficient patients (table 3). RbvDR during the first 4 weeks of treatment was requested in 17 patients (40%) and it was more frequent in non ITPA deficient patients (14/48) as compared to patients with any grade ITPA deficiency (3/21), however this difference was not statistically significant (29% vs 14%, p = 0.2). Erythropoietin use was allowed to manage anemia in this study and was overall required in 43 (62%) patients. ITPA deficiency did not impact the frequency of Erythropoietin use: 65% in the no ITPA deficiency group, 58% in the mild ITPA deficiency group and 56% in the moderate ITPA deficiency setting. Blood transfusions were deemed necessary by the investigator in 18 patients (26%): 27% had no ITPA deficiency, 17% had mild ITPA deficiency and 33% had moderate ITPA deficiency. ITPA deficiency therefore was not associated with RbvDR, erythropoietin support or need for blood transfusions (table 3).

**Table 3:** Hemoglobin decline at week 4 of therapy and management of anemia stratified by ITPA deficiency.

	No ITPA Deficiency (n = 48)	Mild ITPA Deficiency (n = 12)	Moderate ITPA deficiency (n = 9)	*p*
Mean Δ Hb week 4 g/dL	3,85	3,07	1,67	<0,0001
RbvDR, n(%)	29 (60%)	7 (58%)	6 (67%)	1*
Erythropoietin use, n(%)	31 (65%)	7 (58%)	5 (56%)	0.6*
Transfusion, n(%)	13 (27%)	2 (17%)	3 (33%)	1*
Treatment discontinuation within week 12, n(%)	10 (21%)	1 (8%)	1 (11%)	0.3*
Discontinuation for severe anemia, n(%)	4 (8%)	1 (8%)	0 (0%)	1*

*No ITPA deficiency vs any ITPA deficiency.

We analyzed several other baseline factors that could impact on anemia management such as female gender, age, cirrhosis, baseline Hb values and eGFR. By multivariate analysis RbvDR was associated with female sex (OR:18,5; 95% CI 2,06–166,6), Epo use with age >60 years (OR: 4,42; 95% CI: 1,25–15,6), while blood transfusions were associated with either female sex (OR:4,86; 95% CI 1,22–19,22) and baseline Hb values (OR 0,46: 95% CI: 0,25–0,87)

### ITPA and virological response rates

Virological response rates during the first 12 weeks of therapy are resumed in table 4. No impact of ITPA deficiency severity was seen on rates of HCV RNA undetectability at week 4, 8 or 12.

**Table 4:** Rates of HCV RNA undetectability during treatment stratified by ITPA deficiency.

	No ITPA Deficiency (n = 48)	Mild ITPA Deficiency (n = 12)	Moderate ITPA deficiency (n = 9)	*P* *
Undetectable HCV-RNA				
Week 4, n(%)	21 (44%)	6 (50%)	3 (33%)	0,79
Week 8, n(%)	39 (81%)	10 (83%)	9 (100%)	0,48
Week 12, n(%)	38 (79%)	10 (83%)	9 (100%)	0,32

*No ITPA deficiency versus any ITPA deficiency.

## Discussion

Development of severe early anemia during treatment with PR+TVR has been reportedly shown as one of the major clinical issues in patients with advanced fibrosis/cirrhosis. In real life studies focused on this subgroup of patients, grade 3–4 anemia developed in one out of three such patients, requiring Erythropoietin support or blood transfusions in up to 54% and 16% of patients, respectively [3]–[4]. Our cohort of Caucasian patients with advanced fibrosis/cirrhosis confirms these figures, showing that development of early anemia was the main reason for early discontinuation of PR+TVR.

By ITPA genotyping at baseline we were able to show that ITPA polymorphisms could anticipate the severity of Hb decline in the first 4 weeks of PR+TVR, as patients with moderate deficiency were those with a lesser decline in mean hemoglobin values at week 2 and 4 of triple therapy. However, this benefit was lost in the following 8 weeks of triple therapy as the week 12 Hb values were not different across ITPA deficient subgroups. The same finding has been reported by two studies conducted in Japanese patients receiving PR+TVR, which enrolled also patients with mild fibrosis [9]–[10]. Although it is hard to explain why ITPA deficiency protects from development of anemia only in the early weeks of PR+TVR treatment, this might be the direct consequence of the increased plasma Rbv levels that have been reported to occur after 4 weeks of treatment with TVR. In a small study comparing plasma levels of Rbv in 16 patients who received PR and 5 patients who received PR+TVR, intracellular Rbv levels were shown to be similar among the two groups of patients until week 4 of therapy and then increase significantly in the PR+TVR group compared to the PR group [15]. Another small study reported higher Rbv plasma concentrations after 8 weeks of treatment in 9 patients treated with TVR compared to 187 patients who received PR [16].

In theory this findings fit well with the loss of protection from ITPA deficiency towards on treatment anemia that we observed following 4 weeks of PR+TVR therapy. ITPA deficiency is thought to protect against Rbv induced anemia, by protecting against ATP depletion in the erythrocyte. Indeed Rbv has been shown to cause anemia through direct suppression of erythropoiesis as well as due to depletion of GTP which in turn reduces the levels of ATP in erythrocytes [17]. Intracellular ITP, which accumulates in ITPA deficient patients can substitute for erythrocyte GTP, thus allowing physiological levels of ATP in erythrocytes [18]. This explains why at standard doses of Rbv, patients with ITPA deficiency show less severe anemia when treated with PR, however, whether this mechanism still protects from anemia at higher plasmatic Rbv concentrations needs to be demonstrated. High intracellular levels of Rbv in the second and third month of PR/TVR could explain why Rbv reductions have been shown not to impact negatively on SVR rates [19].

Whatever the precise mechanisms by which ITPA deficiency fails to protect from PR+TVR severe anemia may be, our study shows that estimated ITPA deficiency grade through genotyping of *rs1127354* and *rs7270101* at baseline is of limited clinical utility. Indeed, ITPA deficiency was not associated with the management strategy for anemia, as Erythropoietin use, RbvDR and blood transfusion necessity were similar between ITPA groups. This is in our opinion a significant finding as not only it conflicts with what reported for PR therapy, where ITPA deficiency was associated with the need to use erythropoietin and blood transfusions, but also does not support routine genetic testing of ITPA variants before starting PR+TVR [8].

We are aware that our study is not free of limitations, as it was retrospective and conducted on a relatively small sample size of Caucasian patients. The limited sample size of our study was the direct consequence of our choice to stop enrolment after a 6 month period, rather than to conduct an analysis on a predefined number of patients. The rationale behind this was to allow our study to enter the evolving HCV treatment field as quickly as possible, hence making it easily and rapidly accessible for everyday's clinical practice. Still we are aware that this somewhat limits the overall impact of our findings as we cannot completely rule out a Type II error in our analysis, the power of our study to assess the role of ITPA variants on Hb decline was 90.8%. We also acknowledge that, although the anemia management strategy was homogenous between centres, ultimately the choice to reduce Rbv dose, administer Erythropoietin or blood transfuse was at the discretion of the investigator. This was the consequence of the general lack of consensus on how to manage anemia during TVR treatment. Several retrospective studies have shown that RbvDR can be effective in improving anemia without compromising SVR rates, still at this time there are no consensus guidelines on the optimal timing of RbvDR as well as the lowest effective Rbv dose, especially in patients with advanced fibrosis receiving TVR [19–20–21]. Moreover, although RbvDR are considered by the experts to be the best strategy to manage severe anemia during TVR, in some cases severe anemia is so quick to develop that erythropoietin and blood transfusion are necessary to improve the patients quality of life while continuing PR+TVR treatment. Lastly we do not think that our study findings can be translated to the other currently available NS3 protease inhibitor BOC, which also causes anemia through mechanisms that are still poorly understood [21]–[22].

Still all these limitations notwithstanding, we think that our study has clinical relevance as it demonstrates, in a time of limited economical resources, that the clinical utility of ITPA genotyping in HCV-1 patients receiving PR+TVR is limited at least in those with advanced fibrosis/cirrhosis, as it does not predict management of anemia nor development of grade 3 anemia during TVR therapy. Although ITPA deficiency was associated with HB decline during the first 2–4 weeks of therapy, from a clinical standpoint we think this does not support routine ITPA genetic testing in patients candidate to TVR treatment.

## References

[1] European Association for Study of Liver (2014) EASL Clinical Practice Guidelines: management of hepatitis C virus infection. J Hepatol 60(2): 392–420. 24331294 10.1016/j.jhep.2013.11.003

[2] Aghemo A, Degasperi E, Colombo M (2013) Directly acting antivirals for the treatment of chronic hepatitis C: unresolved topics from registration trials. Dig Liver Dis 45(1): 1–7. 22695478 10.1016/j.dld.2012.05.002

[3] Hézode C, Fontaine H, Dorival, Larrey D, Zoulim F, et al (2013) Triple therapy in treatment-experienced patients with HCV-cirrhosis in a multicentre cohort of the French Early Access Programme (ANRS CO20-CUPIC) - NCT01514890. J Hepatol 59(3): 434–41. 23669289 10.1016/j.jhep.2013.04.035

[4] Colombo M, Fernández I, Abdurakhmano D, Ferreira PA, Strasser SI, et al.. (2013) Safety and on treatment efficacy of telaprevir: the early access program for patients with advanced hepatitis C. Gut DOI: 10.1136/gutjnl-2013-305667. 10.1136/gutjnl-2013-305667 PMC4078754 24201995

[5] Werner CR, Franz C, Egetemeyr DP, Malek NP, Laueret UM, et al.. (2013) Efficacy and safety of telaprevir (TVR) triple therapy in a ‘real-life’ cohort of 102 patients with HCV genotype 1: interim analysis after 24 weeks of treatment. J Viral Hepat DOI: 10.1111/jvh.12145. 10.1111/jvh.12145 24716636

[6] Maasoumy B, Port K, Markova AA, Serrano BC, Rogalska-Taranta M, et al (2013) Eligibility and safety of triple therapy for hepatitis C: lessons learned from the first experience in a real world setting. PLoS One 8(2): e55285. 23383319 10.1371/journal.pone.0055285 PMC3562338

[7] Thompson AJ, Fellay J, Patel K, Tillmann HL, Naggie S, et al (2010) Variants in the ITPA gene protect against ribavirin-induced hemolytic anemia and decrease the need for ribavirin dose reduction. Gastroenterology 139(4): 1181–9. 20547162 10.1053/j.gastro.2010.06.016 PMC3086671

[8] Clark PJ, Aghemo A, Degasperi E, Galmozzi E, Urban TJ, et al (2013) Inosine triphosphatase deficiency helps predict anemia, anemia management and response in chronic hepatitis C therapy. J Viral Hepat 20(12): 858–66. 24304455 10.1111/jvh.12113

[9] Ogawa E, Furusyo N, Nakamuta M, Kajiwara E, Nomura H, et al (2013) Clinical milestones for the prediction of severe anemia by chronic hepatitis C patients receiving telaprevir-based triple therapy. J Hepatol 59(4): 667–74. 23707372 10.1016/j.jhep.2013.05.017

[10] Suzuki F, Suzuki Y, Akuta N, Sezaki H, Hirakawa M, et al (2011) Influence of ITPA polymorphisms on decreases of hemoglobin during treatment with pegylated interferon, ribavirin, and telaprevir. Hepatology 53(2): 415–21. 21246582 10.1002/hep.24058

[11] Bedossa P, Poynard T (1996) An algorithm for the grading of activity in chronic hepatitis C. The METAVIR Cooperative Study Group. Hepatology 24(2): 289–93. 8690394 10.1002/hep.510240201

[12] Sandrin L, Fourquet B, Hasquenoph JM, Yon S, Fournier C, et al (2003) Transient elastography: a new noninvasive method for assessment of hepatic fibrosis. Ultrasound Med Biol 29(12): 1705–13. 14698338 10.1016/j.ultrasmedbio.2003.07.001

[13] Castéra L, Vergniol J, Foucher J, Le Bail B, Chanteloup E, et al (2005) Prospective comparison of transient elastography, Fibrotest, APRI, and liver biopsy for the assessment of fibrosis in chronic hepatitis C. Gastroenterology 128(2): 343–50. 15685546 10.1053/j.gastro.2004.11.018

[14] Galmozzi E, Facchetti F, Degasperi E, Aghemo A, Lampertico P (2013) Allelic inhibition of displacement activity: a simplified one tube allele-specific PCR for evaluation of ITPA polymorphisms. J Virol Methods 187(2): 271–3. 23201294 10.1016/j.jviromet.2012.11.026

[15] Hammond K, Jimmerson L, MacBrayne C, Ray M, Bushman L, et al (2013) Increased plasma and intracellular ribavirin concentrations associated with telaprevir use. Rev Antiv Ther Infect dis 3: 26.

[16] Boglione L, De Nicolò A, Cusato J, Cariti G, Di Perri G, et al (2014) Significant early higher ribavirin plasma concentrations in patients receiving a triple therapy with pegylated interferon, ribavirin and telaprevir. J Viral Hepat 21(4): 260–3. 24597694 10.1111/jvh.12170

[17] Fellay J, Thompson AJ, Ge D, Gumbs CE, Urban TJ, et al (2010) ITPA gene variants protect against anemia in patients treated for chronic hepatitis C. Nature 464(7287): 405–8. 20173735 10.1038/nature08825

[18] Hitomi Y, Cirulli ET, Fellay J, McHutchison JG, Thompson AJ, et al (2011) Inosine triphosphate protects against ribavirin-induced adenosine triphosphate loss by adenylosuccinate synthase function. Gastroenterology 40(4): 1314–21. 10.1053/j.gastro.2010.12.038 21199653

[19] Sulkowski MS, Roberts S, Afdhal N, Dusheiko G, Di Bisceglie AM, et al (2011) Ribavirin dose modification in treatment-naïve and previously treated patients who received telaprevir combination treatment: No impact on sustained virologic response in phase 3. J Hepatol 56: S459–S460.

[20] Romero-Gómez M, Berenguer M, Molina E, Calleja JL (2013) Management of anemia induced by triple therapy in patients with chronic hepatitis C: challenges, opportunities and recommendations. J Hepatol 59(6): 1323–30. 23867320 10.1016/j.jhep.2013.07.014

[21] Sulkowski MS, Poordad F, Manns MP, Bronowicki JP, Rajender Reddy K, et al (2013) Anemia during treatment with peginterferon Alfa-2b/ribavirin and boceprevir: Analysis from the serine protease inhibitor therapy 2 (SPRINT-2) trial. Hepatology 57(3): 974–84. 23081753 10.1002/hep.26096

[22] Poordad F, Lawitz E, Reddy KR, Afdhal NH, Hézode C, et al (2013) Effects of Ribavirin Dose Reduction vs Erythropoietin for Boceprevir-Related Anemia in Patients with Chronic HCV Genotype 1 Infection-a Randomized Trial. Gastroenterology 145(5): 1035–1044. 23924660 10.1053/j.gastro.2013.07.051

